# Differential Impact of Membrane-Bound and Soluble Forms of the Prognostic Marker Syndecan-1 on the Invasiveness, Migration, Apoptosis, and Proliferation of Cervical Cancer Cells

**DOI:** 10.3389/fonc.2022.803899

**Published:** 2022-01-27

**Authors:** Katharina Hilgers, Sherif Abdelaziz Ibrahim, Ludwig Kiesel, Burkhard Greve, Nancy A. Espinoza-Sánchez, Martin Götte

**Affiliations:** ^1^ Department of Gynecology and Obstetrics, Münster University Hospital, Münster, Germany; ^2^ Department of Zoology, Faculty of Science, Cairo University, Giza, Egypt; ^3^ Department of Radiotherapy-Radiooncology, Münster University Hospital, Münster, Germany

**Keywords:** syndecan-1, proteoglycan, cervical cancer, shedding, prognosis, metastasis

## Abstract

Cervical cancer ranks fourth among the most commonly diagnosed malignant tumors in women worldwide. Previously published evidence suggested a possible connection between the expression of the membrane-bound heparan sulfate proteoglycan syndecan-1 (Sdc-1) and the development of cervical carcinoma. Sdc-1 serves as a matrix receptor and coreceptor for receptor tyrosine kinases and additional signaling pathways. It influences cell proliferation, adhesion, and migration and is seen as a modulator of the tumor microenvironment. Following proteolytic cleavage of its extracellular domain in a process called shedding, Sdc-1 can act as a paracrine effector. The loss of Sdc-1 expression is associated with low differentiation of cervical carcinoma and with an increased rate of lymph node metastases. Here, we analyzed the clinical impact of Sdc-1 expression by analysis of public gene expression datasets and studied the effect of an overexpression of Sdc-1 and its membrane-bound and soluble forms on the malignant properties of the human cervical carcinoma cell line HeLa through functional analysis. For this purpose, the HeLa cells were stably transfected with the control plasmid pcDNA3.1 and three different Sdc-1-DNA constructs,encoding wild-type, permanently membrane-bound, and constitutively soluble Sdc-1. In clinical specimens, Sdc-1 mRNA was more highly expressed in local tumor tissues than in normal and metastatic cervical cancer tissues. Moreover, high Sdc-1 expression correlated with a poor prognosis in Kaplan-Meier survival analysis, suggesting the important role of Sdc-1 in the progression of this type of cancer. *In vitro*, we found that the soluble, as well as the permanently membrane-bound forms of Sdc-1 modulated the proliferation and the cell cycle, while membrane-bound Sdc1 regulated HeLa cell apoptosis. The overexpression of Sdc-1 and its soluble form increased invasiveness. *In vitro* scratch/wound healing assay, showed reduced Sdc-1-dependent cell motility which was linked to the Rho-GTPase signaling pathway. In conclusion, in cervical cancer Sdc-1 modulates pathogenetically relevant processes, which depend on the membrane-association of Sdc-1.

## Introduction

Cervical cancer represents a major public health problem worldwide. More than 500,000 new cases and approximately 250,000 deaths are reported each year, making cervical cancer the fourth most common type of cancer in women (1). The persistence of Human Papilloma Virus infection is the main factor driving pre-neoplastic lesions and increased risk of cervical cancer, however infection alone is not sufficient to cause cancer (2). Evidence suggests that the microenvironment plays a very important role in the development and progression of cervical cancer. For example, immunosuppression is mediated by the adenosinergic pathway and the presence of immunomodulatory mesenchymal stromal cells (3, 4). Also, the remodeling of the extracellular matrix (ECM) by fibroblasts through the production of laminin is important for cervical cancer cell invasion (5). On the other hand, the ECM receptor Syndecan-1 (Sdc-1) is differentially expressed in cervical intraepithelial neoplasias and carcinoma *in situ* (6). This cell surface heparan sulfate proteoglycan is one of four members of the Sdc family expressed in mouse and human tissues (7). Sdc-1 has well-documented roles in regulating inflammation by modulating the expression and activity of cytokines, chemokines, growth factors, and adhesion molecules through its heparin-related heparan sulfate chains, thus functioning as a signaling co-receptor for different signaling pathways including Rho, Wnt, Hedgehog, Notch, and STAT3 (8, 9). The Sdc-1 protein contains a cytoplasmic, an extracellular, and a hydrophobic transmembrane domain. The short highly conserved cytoplasmic domain mediates oligomerization, binding to Type 2 PDZ domains, intracellular interactions with cytoskeleton, and modulation of signal transduction (9). The extracellular domain harbours attachment sites for HS and can be substituted with chondroitin sulfate chains (7). With the extracellular domain Sdc-1 acts as a matrix receptor for collagen, fibronectin, and laminin isoforms. Notably, Sdc ectodomains can be shed by proteolytic cleavage mediated by a variety of proteases, including matrix metalloproteinases (MMPs), ADAMs, and gamma-secretase, resulting in the conversion of the membrane-bound molecule into a soluble paracrine effector (9). In cancer, Sdc-1 is involved in the regulation of cell migration, cell-cell and cell-matrix interactions, growth-factor, chemokine, and integrin activity, and in the regulation of protease activity (7, 9–11). Importantly, several preclinical and clinical studies have demonstrated that therapies targeting Sdc-1 can inhibit the aggressive behavior of tumor cells (7, 12). Thus, this protein has emerged as a novel target for the development of selective and more potent therapies. Although some studies indicate that Sdc-1 can act both anti- or pro-tumorigenic (13–17), the mechanisms by which Sdc-1 participates in the pathogenesis and progression of tcervical cancer are still unknown. In this study, we analyzed the dysregulation and prognostic impact of Sdc-1 expression in clinical specimens of cervical cancer utilizing publically available transcriptomic datasets. Then, we analyzed the role of Sdc-1 in the proliferation, cell cycle, migration, and invasion characteristics of the well-established cervical cancer cell model line HeLa. Importantly, we studied the individual contributions of membrane-bound and soluble Sdc-1 forms in these processes and the relation between Sdc-1 and RhoGTPases in the invasive characteristics of HeLa cells. Understanding the mechanisms by which different forms of Sdc-1 promote these processes could help to better understand the behavior of cervical cancer cells and to find specific therapeutic targets.

## Materials and Methods

### TNMplot and KM Plot Analysis

To compare the expression of Sdc-1 between non-tumor tissue, tumor, and metastases, we used the TNMplot online tool https://tnmplot.com/analysis/, accessed on 15 September 2021. This platform uses data generated by gene arrays from the Gene Expression Omnibus of the National Center for Biotechnology Information (NCBI-GEO) or RNA-seq from The Cancer Genome Atlas (TCGA), Therapeutically Applicable Research to Generate Effective Treatments (TARGET), and The Genotype-Tissue Expression (GTEx) repositories. Statistical significance was computed using Mann–Whitney or Kruskal–Wallis tests. False Discovery Rate (FDR) was computed using the Benjamini–Hochberg method. The database contains 56,938 samples, including 33,520 samples from 3180 gene chip-based studies (453 metastatic, 29,376 tumorous, and 3691 normal samples), 11,010 samples from TCGA (394 metastatic, 9886 tumorous, and 730 normal), 1193 samples from TARGET (1 metastatic, 1180 tumorous and 12 normal) and 11,215 normal samples from GTEx (18). Survival analysis was performed using the Pan-cancer database of the KMPlot online tool (19), selecting the Cervical squamous cell carcinoma subset (n=304 patients). The median of gene expression was used as a cutoff (median 23526, expression range 298 – 129355). All of the clinical data of the current study are publicly available and have been reviewed in the original studies, therefore there was no necessity for additional ethical review approval processes. The original datasets are described in references (18, 19).

### Cell Culture and Generation of Stably Transfected Cell Lines

The HeLa cell line was purchased from ATCC/LGC Promochem (Wesel, Germany) and cultured in RPMI (Sigma, cat. No. D8758, Deisenhofen, Germany); containing 10% Fetal Calf Serum (FCS, Biochrom GmbH, Cat. No. S0615, Berlin, Germany) and 1% penicillin/streptomycin (Sigma, cat. No. P433) and maintained in a humidified atmosphere of 5% CO_2_ at 37°C. Cells were stably transfected with a pcDNA3.1 control plasmid (Invitrogen, Karlsruhe, Germany) or a plasmid allowing for the overexpression of wild-type (WT), a constitutively membrane-bound (Sdc-1-388), and a constitutively shed form (Sdc-1-392) of murine Sdc-1 in the vector pReceiver-M02 under control of the cytomegalovirus promoter (RZPD/ImaGenes, Berlin, Germany) as previously described (10, 20). Stable clones were selected using 1 mg/ml G418. HeLa cells were cultured in RPMI-1640 medium containing 10% FCS, 1% penicillin/streptomycin and 600 mg/ml G418 in a humidified atmosphere of 5% CO_2_ at 37°C. Successful transfections were confirmed by qPCR.

### Quantitative Real-Time PCR

Total RNA isolation was performed with OLS RNA Kits (OMNI Life Science GmbH & Co. KG, Hamburg, Deutschland) according to the manufacturer’s instructions, and reverse transcribed into cDNA using the First-strand cDNA Synthesis Kit (Thermo Scientific, cat. No. K1612, Waltham, MA, USA) according to the supplier’s protocols. Quantitative real-time PCR was performed in duplicates for each target gene using Universal TaqManR PCR Mastermix (Applied Biosystems, cat. No. 4305719, Foster City, CA, USA), and gene expression levels were measured in an ABI 7300 Real-time PCR detection system (Applied Biosystems, CA, USA). Gene expression was analyzed using the 2^-ΔΔCT^ method and samples were normalized to the expression of *18SRNA*. The ID of the TaqMan probes are: *18srRNA* (hs99999901s1, bp 604, Gen bank accession no. X03205.1), Human *Sdc-1* (hs00174579m1, bp 317 exon boundary 1-2, Gen bank mRNA AJ551176.1), Mouse *Sdc-1* (Mm00448918m1,bp 429, exon boundary 2-3, Gen bank mRNA AK132236.1), *MMP2* (hs00234422m1, bp 1793, exon boundary 12-13, Gen bank mRNA AK301536.1), *ECAD* (hs00170423m1, bp 451, exon boundary 3-4, Gen bank mRNA AB025105.1), *TIMP1* (hs00171558_m1, bp 515, exon boundary 5-6, Gen bank mRNA A10416.1), *BAK* (hs00832876g1 bp 1330, exon 6, Gen bank mRNA AK091807.1), *Bad* (hs00188930m1, bp 188, exon boundary 1-2, Gen bank mRNA AB451254.1), and *BCL2* (hs00153350m1, bp 977, exon boundary 2-3, Gen bank mRNA BC027258.1) (Assays by Applied Biosystems, CA, USA).

### Cell Proliferation Assay

Cell proliferation was evaluated using alamarBlueR-System (Thermo Scientific, cat. No. A50100). A total of 5000 cells/well were seeded in 96-well plates and maintained in RPMI medium supplemented with 10% FCS. After 24 h, 20µl alamarBlueR-substrate was added. Following the manufacturer’s protocol, after 6 h the colorimetric change was analyzed.

### Cell Cycle Analysis

For DAPI staining, cell pellets were resuspended in 1 mL of 4,6-diamidino-2-phenylindole (DAPI) (CyStain UV Ploidy, cat. no. 05-5001, Sysmex, Norderstedt, Germany) and after 5-min incubation at RT, cells were analyzed by flow cytometry (CyFlow space, Sysmex/Partec, Münster, Germany). Excitation was carried out with a 375-nm UV laser and fluorescence emission was measured at 455 nm in FL4. Data analysis was performed with FlowJo software (LLC).

### Apoptosis Assay

Cells were stained with the Annexin V/propidium iodide (PI) (Thermo Fisher Scientific, cat. No. V13242), as detailed by the manufacturer and as previously described (21). Measurement was performed on a flow cytometer using FloMax software (Quantum Analysis, Münster, Germany) to visualize and manage flow data. For interpretation, the fourth quartile in the measurement graph indicated apoptotic cells, where cells are positive for Annexin V. Annexin V binds to phosphatidylserine when cell membranes lose lipid asymmetry during apoptosis, but are negative for propidium iodide as cell membranes remained intact (22).

### Invasion Assay

Transfected cells were diluted to 50.000 cells/mL in RPMI media containing serum. Then, 500 μL (corresponding to 25.000 cells) were transferred to Matrigel-coated inserts (Corning^®^, cat. no. 354230; Bedford, MA). This was followed by a 24-hour incubation period at 37°C, 7.5% CO_2_. After carefully removing RPMI media and replaces by 500 μL of RPMI without serum, the invasion was triggered by adding 750 μL of RPMI medium with FCS as a chemoattractant factor into the lower compartment of the chamber. After 24 hours, all media was removed, and the cells on top of the Matrigel were removed with “cotton-wool” sticks and washed in PBS for 1 minute. After removing the PBS, the cells were stained in 1% toluidine blue in BORAX (Sigma, cat. No. T3260) for 6 minutes and washed with H_2_O. Two non-overlapping pictures were taken under a Zeiss Axiophot (Zeiss, Jena, Germany) bright-field microscope (magnification 10X) and invaded cells were counted.

### Wound Healing Assay

Cells were cultivated in 6-well plates for 72 hours in triplicate. After that, the cells were washed one time with 1X PBS, and then a scratched area was created using a sterile 200 μL pipette tip on 90% confluence, followed by incubation in serum-free RPMI medium for 24 hours. Cells migrated into the wound surface were determined under the microscope at time intervals of 0, 6, 8.5, and 23 hours. Images of scratched areas were captured with Zeiss Axiophot bright-field microscope (magnification 10X). The ratio of cell migration was calculated as the percentage of the remaining cell-free area compared with the area of the initial scratched area using the Axio Vision program (Carl Zeiss Microscopy GmbH, Göttingen, Germany). With a 10 X scaling, a contour was drawn around the cells at the edge of the scratch and the wound area was calculated in μm^2^, supported by the software. Some assays were performed in the presence or absence of 10 μM of the Rho kinase (ROCK) inhibitor Y-27632 (Stem Cell Technologies, cat. No. 72302).

### Immunofluorescence Microscopy and Immunocytochemistry

Cells (25,000) were cultured in 8-well slides. After the cells adhered, the medium was removed completely, and then, cells were fixed with 3.7% paraformaldehyde (Merck KGaA, K42464803, Darmstadt, Germany) for 10 minutes, followed by a 5-minute incubation in 0.1% Triton X-100 (Carl Roth GmbH and Co. KG, 3051.3, Austria) to allow permeabilization. Cells were washed twice with 1X PBS for 5 minutes each and incubated for 30 minutes with 10% Aurion BSA (AURION, 60613/3, Wageningen, Netherlands). For immunofluorescence microscopy, the cells were incubated with the primary Rabbit-anti-human RhoB (Santa Cruz Biotechnology, Santa Cruz, California, USA) in Dako antibody diluent (1:50, Agilent Technologies, S2022, California, USA) for 1 hour at room temperature (RT). After that, cells were incubated for 30 min at RT with the secondary IgG anti-rabbit conjugated with AlexaFluor 488 (cat. No. A48282, Thermo Fisher Scientific, USA) diluted at 1:600 in DAKO antibody diluent. For staining of actin filaments, cells were incubated for 30-minutes with Phalloidin CruzFluor™ 594 Conjugate 1000X (1:500, Santa Cruz Biotechnology, cat. No. sc-363795, Texas, USA) in Dako antibody diluent. Then, samples were washed three times with PBS for 5 minutes each and incubated with DAPI diluted 1:5000 in PBS for an additional 1 minute, followed by rinsing with PBS. Coverslips were mounted with Vectashield Medium (Vector Laboratories, Cat. No. H-1000-10, California, USA). Samples were examined by fluorescence microscopy (Olympus BX 61, Camera Regina 4000 R). The image sections were reproduced at 400X magnification using the Q Capture 2.73.0 Media Cybernetics Image Pro^®^ software (Bethesda, USA). For each cell line, several images were photographed from the three independent experiments. For immunocytochemistry of murine Sdc-1, cells were cultured and processed analogously up to the stage of primary antibody incubation. Cells were then stained with rat-anti-mouse Sdc-1 mAb 281-2 (BD Pharmingen, San Jose, CA, 1:1000 in PBS/1% bovine serum albumin (BSA)), Endogenous peroxidase was quenched with methanol/0.6% H_2_O_2_, followed by three washes with PBS. Murine Sdc-1 was detected using the Vectastain ABC kit (anti-rat), Vector Laboratories Inc., Burlingame, CA), and the AEC substrate (DAKO, Glostrup, Denmark), followed by counterstaining with Mayer’s Hemalum (Merck, Darmstadt, Germany). Sections were observed with a Zeiss Axiovert 100 microscope equipped with an Axiophot Mrc camera.

### Flow Cytometry for Syndecan-1

To detect cell surface Sdc-1, HeLa cells were detached using 1.5 mM ethylenediaminetetraacetic acid (EDTA) in Ca/Mg-free PBS buffer for 10 min at 37°C with gentle agitation. Cells were washed in PBS and resuspended in cold buffer containing 1% FCS. A total of 2 x 10^5^ cells per sample were used for a single analysis. Following centrifugation, cells were resuspended in PBS/2% BSA and incubated for 15 min at 25°C with 10 μl of anti-human Sdc-1 (CD138)-PE (eBioscience, Inc., San Diego, CA, USA) or an isotype control IgG. Stained cells were analyzed by a cube-8 flow cytometer (Sysmex/Partec, Muenster, Germany).

### Dot Blot Assay for Shed Syndecan-1

To detect soluble (shed) human and murine Sdc-1, cell culture supernatants were collected from the transfected HeLa cell lines grown for 5 days in 75 cm² flasks (12 ml medium, 6 x 10^6^ cells at time of harvesting). 600µl of cleared (10 000g, 4°C, 10 min) cell culture supernatants were loaded on nitrocellulose membranes using a microfiltration apparatus (Bio-Rad, Hercules, CA). Membranes were blocked for 60 min with 3% non-fat dry milk, 0.5% bovine serum albumin, 150 mM NaCl, 50 mM Tris-HCl, pH 7.4, and incubated for 16 h at 4°C with the rat-anti-mouse Sdc-1 antibody 281-2 (BD Pharmingen, San Jose, CA 1:100 in PBS/1% BSA) at 4°C or the mouse anti-human Sdc-1 antibody DL-101 (1:100 in PBS/1%BSA, Santa Cruz Biotechnology, Santa Cruz, CA, USA). Immunoreactivity was visualized with HRP–conjugated secondary antibodies (1:5000 in PBS/1%BSA, Sigma, Deisenhofen, Germany). Antibodies were detected by enhanced chemiluminescence according to the manufacturer’s instructions using SuperSignal™West Pico PLUS Chemiluminescent Substrate (Thermo Scientific™, cat. no. 34580, Foster City, CA, USA) in a FUSION SL (Vilber Lourmat, Marne-la-Vallée Cedex, France) device. Digitalized images were analyzed densitometrically using ImageJ software (National Institutes of Health, Bethesda, MD), and expressed as percentage of the vector control cells.

### Statistical Analyses

Statistical analysis of *in vitro* data was performed with GraphPad Prism 4.02 (GraphPad Software, La Jolla, CA). GraphPad Prism 4.02 was used to perform two-tailed t-tests, one-way ANOVA with Dunn´s posttest, or nonparametric Friedman’s test with Dunn’s posttest, where appropriate. Data of the TNM Plotter resource were analysed using Mann-Whitney Test or Kruskal–Wallis test as appropriate, whereas data of the KM Plotter resource were analysed by Cox proportional hazards regression analysis calculatinge log-rank P values, and hazard ratios (HR) with the “survival” R package v2.38. Data were considered significant, when P-values were below 0.05.

## Results

### Sdc-1 Is Highly Expressed in Cervical Carcinoma, and Correlates With a Poor Overall Survival

**Figure 1:**
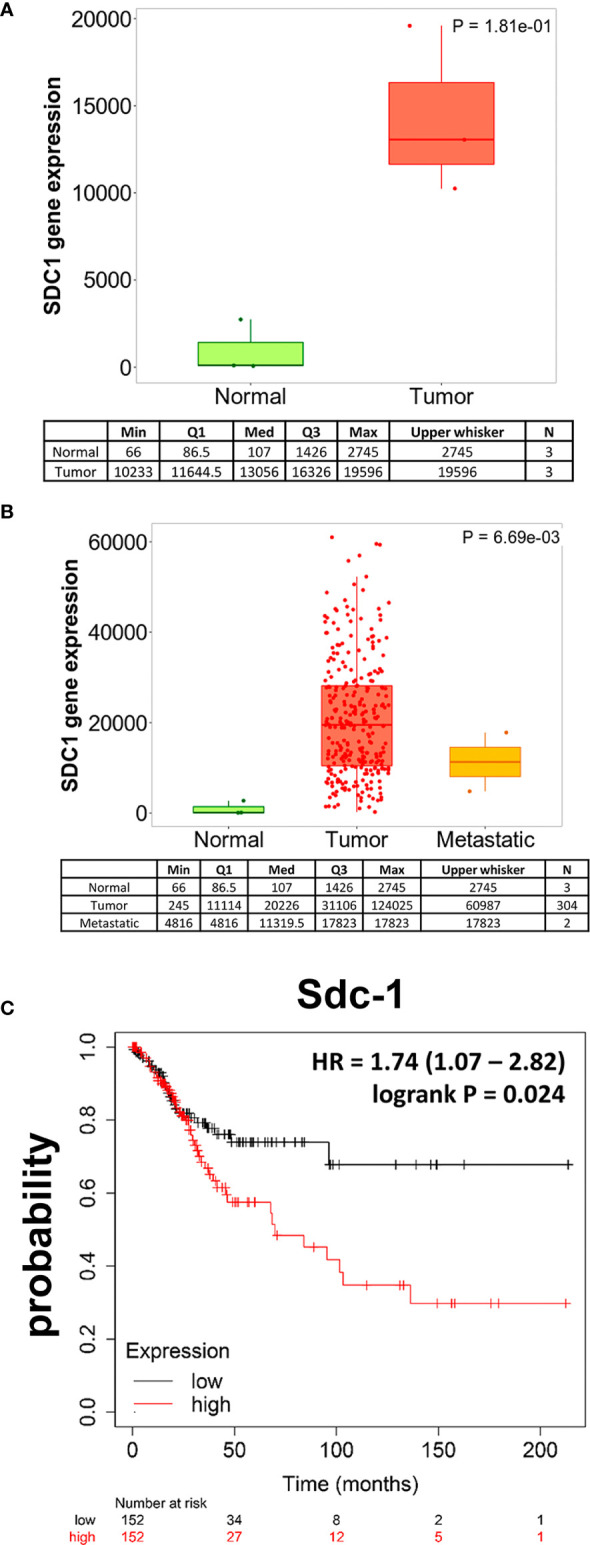
Sdc-1 is dysregulated in cervical carcinoma, and its high expression correlates with poor overall survival. Boxplots of SDC-1 gene expression in cervical cancer tissue when comparing paired normal and tumor RNA Seq data of three patients. **(A)** and when comparing normal, tumor and metastasis RNA Seq data **(B)**. The quantile cutoff values (minimum, 1st quartile, median, 3rd quartile, maximum) and the number of analyzed samples are presented. **(C)** Kaplan-Meier Survival analysis of 304 cervical carcinoma patients stratified by high or low expression of Sdc-1. **(A)** p=0.181 (not significant), Mann-Whitney-Test. **(B)** p=0.069 (Kruskal-Wallis test, trend for significance), *post hoc*-analysis p-values (Dunn’s test): p=1.65x10^-03^ normal tissue compared to tumors (significant), p=0.175 tumor tissue compared to metastases (not significant), p=0.115 normal tissue compared to metastatic tissue. **(C)** p=0.024 (significant), Cox regression analysis log rank p-value.

Previous studies have indicated a dysregulation of Sdc-1 expression in cervical carcinoma tissues (6, 13–17). To further investigate the clinicopathological relevance of aberrant Sdc-1 expression in this disease, we made use of large public gene expression datasets. Using the RNA Seq-based gene expression data of the TNMplot online tool (18), we found that the expression of Sdc-1 in 3 paired samples of adjacent normal cervical tissue cervical squamous cell carcinoma and endocervical adenocarcinoma was strongly, yet non-significantly upregulated in the malignant tissue (**Figure 1A**). Further analysis of a larger number of cancerous tissues (n=304) and metastases (n=2) showed a trend for a higher expression compared to normal tissues (n=3) (p=0.0669, Kruskal-Wallis-test) (**Figure 1B**). Post-hoc analysis by Dunn test revealed a p-value of 1.65x10^-03^ for the comparison of normal tissue and tumors, a p-value of 1.75x10^-01^ comparing tumor tissue and metastases, and a p-value of 1,15x10^-01^-for the comparison of normal and metastatic tissue. We next utilized gene chip-based mRNA expression data of 304 cervical carcinoma patients and correlated the high vs low expression of Sdc-1 with patient survival using the cervical carcinoma subset of the KM Plotter pan-cancer database (19). Using the median as cutoff, a high expression of Sdc-1 was found to significantly correlate with a poor overall survival (HR = 1.74 (1.07 − 2.82), logrank P = 0.024) (**Figure 1C**). Overall, these data confirm previous evidence for a dysregulation of Sdc-1 expression in cervical cancer, and provide novel evidence for its utility as a prognostic marker in mRNA-based analyses.

### Overexpression of Membrane-Bound and Soluble Sdc-1 Affects Proliferation and the Cell Cycle, While Membrane-Bound Sdc-1 Increases Apoptosis of HeLa Cells

**Figure 2:**
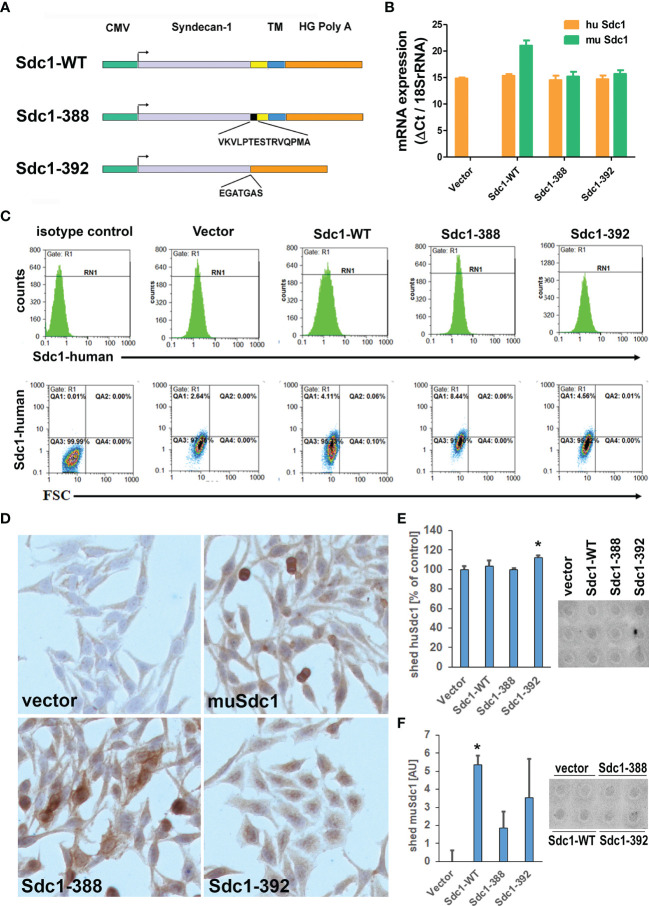
Characterisation of HeLa cells overexpressing wild-type, constitutively membrane bound (Sdc-388) and constitutively shed (Sdc1-392) Sdc-1. **(A)** Schematic representation of the plasmid Sdc-1 cDNA inserts, CMV, cytomegalovirus promoter; yellow box, juxta-membrane domain; Black box, non-cleavable CD4 sequence; blue box, transmembrane and cytoplasmic domain, orange box, poly-A-tail; Sdc1-WT murine wild-type form; Sdc1-388, uncleavable construct 388;Sdc1-392, constitutively shed construct 392. **(B)** Quantitative PCR analysis of murine (mu Sdc-1 and human (Hu Sdc-1) Sdc1 expression. Sdc-1 expression was related to the housekeeping gene 18SrRNA. *n* ≥ 3, error bars = SEM. **(C)** Detection of human Sdc-1 protein expression at the surface of the transfected HeLa cell lines using flow cytometry. Cells were stained for isotype control mouse IgG1-PE and mouse anti-human Sdc-1 (CD138)-PE and the cells were subjected to flow cytometry. Human Sdc-1 is expressed at the cell surface of all cell types. **(D)** Immunocytochemistry for murine Sdc-1, demonstrating expression of murine Sdc-1 in Sdc1-WT, Sdc-1-388 and Sdc-1 392 transfected cells (brown-red staining). Original magnification 10x. **(E, F)** Detection of shed human **(E)** and murine **(F)** Sdc-1 in cell culture supernatants of the transfected cell lines. Conditioned media were collected from the cell lines indicated and 600 µl were subjected to a dotblot assay and quantified by Image J densitometric analysis. Left panels = quantification, right panels= representative dot-blots, n>3, *= p<0.05 Sdc1-392 compared to vector control (t-test). The cell lines shed comparable amounts of human Sdc-1 into the culture media, with a moderately, yet significantly enhanced amount in Sdc1-392 cells. Shed amounts of murine Sdc-1 were variable, with Sdc1-WT cells showing significantly increased levels of shed murine Sdc-1 compared to vector control (n>3, *p<0.05, t-test).

Previous studies using heterologous overexpression of membrane-bound and soluble forms of murine Sdc-1 in human breast cancer cells had provided valuable insights on their differential role in invasive growth of breast cancer cells (10). To understand the roles of the wild type (Sdc1-WT), membrane-bound (Sdc1-388), and soluble Sdc-1 (Sdc1-392) in the progression of cervical cancer, the human cervical cancer cell line HeLa was stably transfected with three different Sdc-1 DNA constructs and a control vector (**Figure 2A**), as previously described (10). The control plasmid pcDNA3.1 does not contain an insert. The Wild-Type Sdc-1 plasmid overexpresses murine Sdc-1 under the strong CMV promoter. Murine and human Sdc-1 share 70% amino acid sequence identity in their extracellular, 96% in their cytoplasmic, and 100% in their transmembrane domains (10, 23). The construct Sdc1-392 encodes only the extracellular domain of Sdc-1 and the construct Sdc1-388 enables the overexpression of a constitutively membrane-bound (non-cleavable) form, in which the shedding site is replaced by CD4 sequences (10). To verify overexpression of Sdc-1, the expression of murine Sdc-1 constructs was quantified by qRT-PCR. As expected, the control plasmid did not express murine Sdc-1 (**Figure 2B**). We confirmed a slight overexpression of the murine Sdc1-WT, while the constitutively membrane-bound and soluble murine Sdc-1 were detected at almost the same levels as the endogenous human Sdc-1 (**Figure 2B**). Therefore, these results confirm that the transfection was successful, and that the altered Sdc-1 constructs were ectopically expressed at levels comparable to endogenous human Sdc-1. We next confirmed the presence of human Sdc-1 protein at the cell surface of the transfected cells using flow cytometry (**Figure 2C**). In all cell lines, human Sdc-1 levels clearly exceeded background expression levels, with cells expressing the heterologous constructs exceeding the levels of the vector control (2.64% vs 4.11-8,44%). We next studied the expression of the heterologous murine constructs by immunocytochemistry (**Figure 2D**). Vector control cells showed only a neglible background staining for murine Sdc-1. Murine Sdc-1 was distributed at the cell surface and cytoplasm of WT-Sdc1 cells, and showed a more pronounced membranous staining in Sdc1-388 cells. Cytoplasmic staining in these cells may mark passage through the secretory pathway. In contrast, no membranous murine Sdc-1 staining was seen in Sdc1-392 cells, where cytoplasmic and nuclear staining of Sdc-1 were observed (**Figure 2D**). To study the levels of soluble, shed Sdc-1 in our cell models, we employed a dot-blot analysis of conditioned media (**Figures 2E, F**). Even after long exposure times, signals of shed Sdc-1 were only slightly detectable above background levels, demanding cautious interpretation of the results. All cells shed comparable amounts of human Sdc-1 into the media, with a slight (approx. 10%), yet significant increase noted in cells overexpressing shed murine Sdc-1 (Sdc1-392) (**Figure 2E**). With respect to murine Sdc-1, results showed greater variability. While both Sdc1-WT and Sdc1-392 cells showed clearly increased soluble murine Sdc-1 levels over vector controls, the increase was only significant in the case of Sdc1-WT cells **(** **Figure 2F**). Overall, the results demonstrate the presence of membrane-bound and soluble human Sdc-1 on our cell models, and a proper expression and localisation of the heterologous murine Sdc-1 constructs over a background of endogenous human Sdc-1.

**Figure 3:**
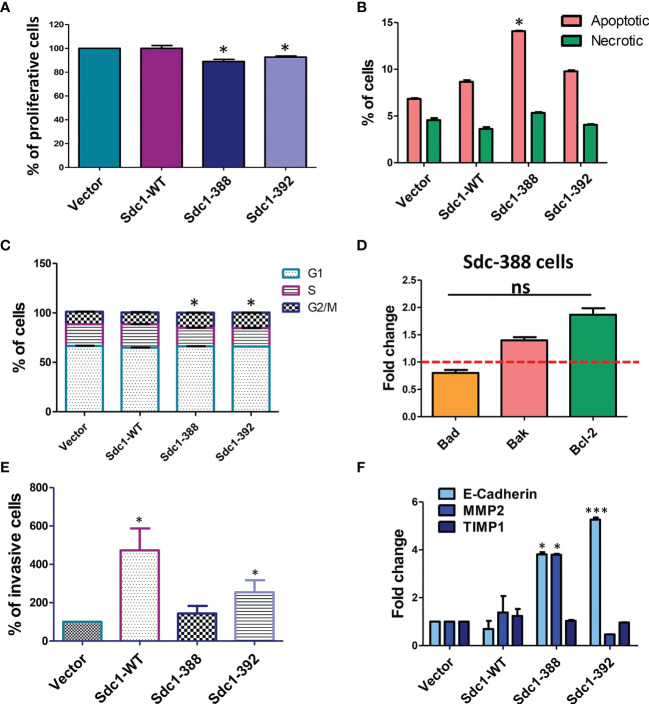
Role for soluble and membrane bound forms of Sdc-1 in HeLa cell proliferation, cell cycle progression, apoptosis and invasion. **(A)** Differential effect of membrane-bound and soluble Sdc-1 on breast cancer cell proliferation. Control vector-transfected HeLa and HeLa cells stably overexpressing WT (Sdc1-WT), constitutively membrane-bound (Sdc1-388) or the soluble ectodomain (Sdc1-392) of Sdc1 were subjected to an Alamar Blue cell proliferation assay *P < 0.05 (One-way ANOVA with Dunn’s *post hoc* test) for Sdc1-388 compared to vector control and Sdc1-392 compared to vector control *n* ≥ 3, error bars = SEM. Changes in apoptosis **(B)** and cell cycle progression **(C)** after the stable transfection of HeLa cells as quantified by using Annexin V/propidium iodide **(B)** and by DNA staining **(C)** and followed by flow cytometry. *P < 0.05 (One-way ANOVA with Dunn’s *post hoc* test) for Sdc1-388 compared to vector control, and Sdc1-392 compared to vector control, *n* ≥ 3, error bars = SEM. **(D)** quantitative Real-Time PCR of the expression of the apoptosis markers Bad, Bak and Bcl-2 in the HeLa Sdc1-388 transfected cells. Data are expressed as fold change versus control vector-transfected cells. ns, no significant p value for all group comparisons (one-way ANOVA with Dunn’s *post hoc* test). n≥3, error bars = SEM. **(E)** Stably transfected HeLa cells were subjected to a matrigel invasion assay. Quantification of invasive cells relative to control vector-transfected cells. *p <0.05 Sdc1-WT compared to vectror controls and SDc1-392 compared to vector controls, (one-way ANOVA with Dunn’s *post hoc* test), n≥4, error bars = SEM. **(F)** quantitative RT-PCR analysis of E-cadherin, MMP2 and TIMP1 compared with vector controls. *p <0.05, MMP2 Sdc1-388 compared to vector controls, E-cadherin Sdc1-388 compared to vector controls, ***p <0.001, E-cadherin Sdc1-392 compared to vector controls (one-way ANOVA with Dunn’s *post hoc* test), n≥3, error bars = SEM.

Due to the capacity of Sdc-1 to act as ligands or co-receptors for various signal-transducing receptors, affecting pathways associated with the hallmarks of cancer, namely proliferation, cell cycle, and apoptosis (24, 25), we performed functional analysis related to these processes. We observed that both soluble (Sdc1-392) and constitutive membrane-bound Sdc-1 (Sdc1-388) moderately inhibited HeLa cell proliferation. Compared with vector controls, membrane-bound Sdc1-388 decreased proliferation to 89% and soluble Sdc-1-392 decreased proliferation to 92%. Murine Sdc1-WT overexpression did not affect proliferation (**Figure 3A**). **Figure 3B** shows that the overexpression of membrane-bound Sdc1-388 affected apoptosis. The percentage of apoptotic cells was 13.9% in membrane-bound Sdc-1-388 cells compared to the control with 6.75%, the soluble Sdc1-392 with 9.67%, and Sdc1-WT with 8.49% (**Figure 3B**). Regarding the cell cycle, the overexpression of soluble Sdc1-392 and membrane-bound Sdc1-388 led to a significant shift of the HeLa cells from the S-phase to the G2M-phase (**Figure 3C**). Approximately, 22% of control cells (vector and Sdc1-WT) are in the S-phase, while 18.6% of membrane-bound Sdc-1-388 and 18.5% of soluble Sdc-1-392 were in the S-phase. Interestingly, a clear shift to the G2M phase was observed from a 12.4% control to a 15.25% of membrane-bound Sdc1-388 and 15.53% of soluble Sdc1-392 in HeLa cells (**Figure 3C**). Since the membrane-bound Sdc1-388 affected apoptosis, we evaluated the expression levels of apoptosis-related genes in the Sdc1-388 HeLa cells such as *Bad*, *Bak*, and *Bcl-2* by qRT-PCR. No significant alteration in gene expression was detected, suggesting that transcriptional changes of these markers as a cause of increased apoptosis can be excluded (**Figure 3D**). These results suggest that both the membrane and soluble forms of Sdc-1 have an impact on proliferation, apoptosis, and the cell cycle in cervical cancer cells.

### Overexpression of Sdc1-WT and Soluble Sdc1-392 Increases Cervical Cancer Cell Invasiveness

Previously, we observed that the depletion of Sdc-1 in breast and colon cancer cells increases their migration and invasion capacity (24, 26), while in endometriotic cells the expression of Sdc-1 promotes their invasive potential (27). Moreover, membrane-bound and Sdc-1-WT promoted invasion of breast cancer cells *in vitro* (11). To analyse a possible role of Sdc-1 in the invasion capacity of the transfected HeLa cells, we performed matrigel invasion assays. Overexpression of Sdc1-WT and soluble Sdc1-392 significantly increased the invasion capacity of HeLa cells (430% and 230%, respectively) compared with vector control cells (100%). Overexpression of membrane-bound Sdc1-388 did not affect invasiveness (**Figure 3E**). To investigate if Sdc-1 in its different forms influences the expression of invasion-related factors, we analyzed the expression levels of *E-cad*, *MMP2*, and tissue inhibitor of metalloproteinases 1 (*TIMP-1*), which are also associated with the metastasis of cervical cancer cells (28, 29) by qRT-PCR. In cells transfected with the membrane-bound Sdc1-388, we observed a significant overexpression in both *E-cad* and *MMP2*. Moreover, *E-cad* was also overexpressed in the Sdc1-392-transfected cells, while no gene expression changes were noted with respect to cells overexpressing Sdc-1-WT (**Figure 3F**).

### Sdc-1 Overexpression Inhibits Migration of HeLa Cells in Rho-GTPase-Dependent Mechanism

**Figure 4:**
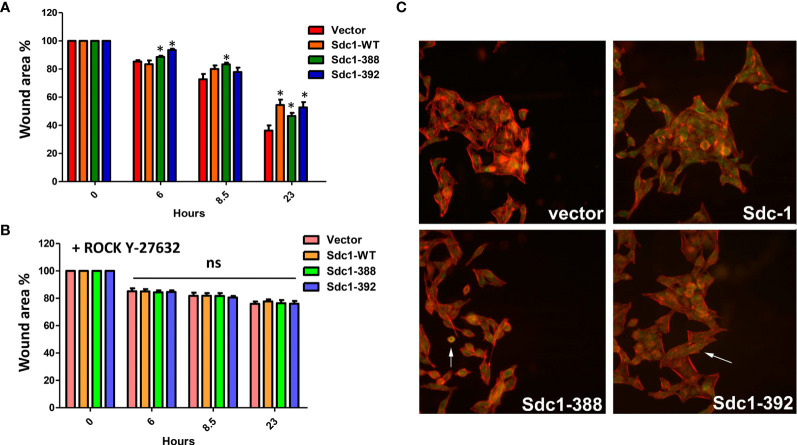
Migration of HeLa cells is decreased by all forms of Sdc-1 in a Rho-GTPase dependent manner. **(A)** The Scratch/wound area of the four cell lines is shown as a percentage of the wound area at time 0h, 6h, 8.5h and 23h. The ability of all three cell constructs to migrate is reduced compared to the control cell line. * = p <0.05 (nonparametric Friedman’s test with Dunn’s posttest) for Sdc1-388 compared to vector control (t=6h, 8.5h, 23h), for Sdc1-WT vs vector control (t=23h) and for Sdc1-392 vs vector control (t=6h, 23h), n≥3, error bars = SEM. **(B)** Stably transfected HeLa cells were treated with the Rho kinase (ROCK) inhibitor Y-27632 and then a Scratch/wound area was created. The area of the three cell constructs and vector was quantified at 0h, 6h, 8.5h and 23h. ns, no significant difference compared to vector control (nonparametric Friedman’s test with Dunn’s posttest). n≥3, error bars = SEM. **(C)** Confocal immunofluorescence microscopic analysis of RhoB protein in HeLa transfected cells. green, RhoB; red, actin-binding protein phalloidin for cytoskeletal staining; blue, DAPI staining for nucleus. Representative images are presented. 40x magnification.

We next performed migration assays to analyze if changes in invasive growth may be linked to an altered migration capacity of the Sdc-1-manipulated HeLa cells. For this purpose, a scratch area was created in the transfected HeLa cells, and after 6, 8, and 23 h closing of the cell-free area was analyzed. At 6 hours, the membrane-bound Sdc1-388 and soluble Sdc1-392 cells showed less migration capacity relative to control and Sdc1-WT cells, while at 8 h only the membrane-bound Sdc1-388 cells were less migratory compared to the other cells (**Figure 4A**). At 23 h the vector cells have a higher migration capacity relative to Sdc1-WT, membrane-bound Sdc1-388, and soluble Sdc1-392 HeLa cells, which displayed a decreased migratory phenotype (**Figure 4A**). These results suggest that the wild-type and the soluble form of Sdc-1 have an impact on the invasion, while all forms of Sdc-1 influence the migration capacity of cervical cancer cells. We previously found that the depletion of Sdc-1 in the triple-negative breast cancer MDA-MB-231 cell line leads to an increase in migration and invasion which was dependent on the expression and activity of Rho-GTPase (24). To decipher the role of Sdc-1 and Rho in the migration capacity of cervical cancer cells, the transfected HeLa cells were cultured in the presence of the specific inhibitor of Rho-Kinases (ROCK) Y-27632. Interestingly, due to the pharmacological blockade of the Rho signaling pathway, the Sdc-1-specific effect on cell migration could be inhibited (**Figure 4B**). This suggests that the Sdc-1-dependent changes in migration of cervical cancer cells depend on the Rho signaling pathway. Finally, since Sdc-1 influences the motility of the HeLa cells in a Rho-dependent manner, we performed immunofluorescence staining to examine the distribution of RhoB in the HeLa-transfected cells. Phalloidin staining demonstrated actin fiber formation that was particularly prominent at the margins of cell groups, demonstrating cytoskeletal remodeling of the cervical cancer cells. Moreover, in all cell lines, RhoB showed a cytoplasmic localisation (**Figure 4C**). However, we found that the overexpression of the soluble Sdc1-392 induced an increased localization of RhoB at the cell-cell boundaries, which indicates an increased membrane localization (**Figure 4C**, arrow), and could influence the activation of the GTPase signal. On the other hand, overexpression of membrane-bound Sdc1-388 induced cell rounding that could indicate the presence of apoptotic cells (**Figure 4C**, arrow), which is in agreement with the result of apoptosis analysis (**Figure 3B**).

## Discussion

Sdc-1 plays an important role in the progression of different types of cancers by regulating the hallmarks of cancer such as proliferation, angiogenesis, apoptosis, invasion, and metastasis (7, 9, 25, 30, 31). Despite all this evidence, little is known about how Sdc-1 affects the progression of cervical cancer. On one hand, it has been observed that the cervix exhibits a differential expression of Sdc-1 depending on the type of cell and epithelium as well as, in non-neoplastic and neoplastic lesions and the histological grade of the tumor (13, 16). Low Sdc-1 expression was observed in the progression of cervical intraepithelial neoplasia (CIN) grade I to grade III, while in poorly differentiated squamous cell carcinomas, Sdc-1 was almost absent (13). Immunohistochemical analysis of cervix tumor tissues showed that the cell surface Sdc-1 expression was higher on stromal fibroblasts than in cancer cells and that patients with high cell surface Sdc-1 expression had significantly better survival (14). In another study, the authors observed that intensity of Sdc-1 staining was higher in the normal epithelium, followed by CIN, and by invasive squamous cell carcinoma (15). Importantly, an inverse correlation between the expression of Sdc-1 in the primary site and lymph node metastasis was observed (15), suggesting that Sdc-1 has a different role in the different stages of development of cervical cancer. In our study, we found that Sdc-1 mRNA showed a trend for a higher expression in local tumor tissues than in normal and metastatic cervical cancer tissues, and a significant correlation with poor survival of cervical carcinoma patients (**Figure 1**). These data suggest an important role of Sdc-1 in the progression of this type of cancer. In a cohort of 124 samples of primary invasive carcinoma of the cervix, a high expression of Sdc-1 was observed in 39% of the samples which was associated with the grade of differentiation and squamous histology but was not associated with the disease-free survival (16). In a different study, from 121 samples of cervical cancer, 101 (83.5%) were positive for Sdc-1 being the histological type, and grade those that showed statistical significance with Sdc-1 expression (32). In this case, high Sdc-1 expression in the cytoplasm was related to better patient survival (32). These results suggest that in cervical cancer, Sdc-1 plays an important role in the development and maintenance of the primary tumor. Therefore, it would be important to carry out more studies that involve a greater number of samples including primary and metastatic tumors. To know more about the role of Sdc-1 in processes associated with malignancy, we analyzed the effect of the overexpression of Sdc-1 and its membrane-bound and soluble form on the malignant properties of the human cervical carcinoma cell line HeLa through functional analysis. We previously showed that in breast cancer cells, the membrane-bound and the soluble form of Sdc-1 exert different functions (10). Here, we found that the soluble, as well as the permanently membrane-bound state of Sdc-1, decreased the proliferation and the cell cycle progression of HeLa cells (**Figures 3A, C**). In breast cancer cells, the overexpression of WT Sdc-1 increased cell proliferation, whereas overexpression of the soluble form Sdc1 inhibits proliferation (10). It has been observed that Sdc-1 shedding potentially affects tumor growth and metastasis (30). This suggests that the soluble form of Sdc-1 has an important role in proliferation, but its role is tumor type-dependent. We also observed that the soluble, as well as the permanently membrane-bound state of Sdc-1, modulated the cell cycle from S to G2/M phase. Interestingly, in mesothelioma Sdc-1 promotes an arrest in the G1 phase by modifying the heparan sulfate composition (33). Regarding apoptosis, in endometrial cells, Sdc-1 expression prevents their apoptosis (34). In agreement, in myeloma Sdc-1 functions as an inhibitory factor of apoptosis. Also, inhibiting IGF1R, which is captured by Sdc-1, a reduction in size and vasculature of myeloma tumor xenografts was observed (35). On the contrary, we here observed that the membrane-bound Sdc1-388 promotes the apoptosis of HeLa cells (**Figure 3B**). Again, the effect of Sdc-1 on apoptosis seems to be dependent on the type of tumor but also on its membrane localisation.

In our study, the overexpression of Sdc-1 and its soluble form Sdc1-392 increased HeLa cell invasiveness (**Figure 3E**). In concordance, soluble Sdc-1 promoted the invasion of breast cancer cells (10). In contrast to our findings in breast cancer cells (10), we observed an increase in the expression of *E-cad* and no changes in TIMP expression in the cells transfected with the soluble form (**Figure 3F**). This suggests that in cervical cancer cells, the soluble form of Sdc-1 has different downstream targets. However, it should be noted that loss of expression of E-cadherin did not affect pancreatic tumor cell motility and metastasis (36). Further, elevated E-cadherin expression enhances invasion and passive dissemination of SUM149 inflammatory breast cancer cells *via* induction of cell-cell adhesion and formation of tumor clusters or emboli (37, 38). Moreover, in head and neck squamous cell carcinoma the cells with the higher levels of Sdc-1 are less migratory and invasive (39), while the increase in soluble Sdc-1 favors migration and angiogenesis in myeloma (40). Interestingly, the progression of CIN to early invasive cervical cancer was associated with low levels of Sdc-1 (17). Distinguishing the role of membrane-bound and soluble Sdc-1 with respect to invasive behavior, it is surprising that MMP2 was downregulated in the most invasive cell type (WT-Sdc-1), whereas it was upregulated in moderately invasive Sdc1-388 cells (**Figure 3F**). We can only speculate if upregulation of MMP2 in Sdc1-388 cells may have been balanced by the upregulation of anti-invasive E-cadherin, which may have also contributed to a weaker pro-invasive effect in the Sdc1-392 cells compared to Sdc1-WT (**Figures 3E, F**). Regarding migration, our *in vitro* scratch/wound healing assay showed reduced Sdc-1-dependent motility of the HeLa cells which was mediated by the Rho-GTPase signaling pathway (**Figure 4**). These results appear counter-intuitive considering the effect of the different forms of Sdc-1 on invasion, but demonstrate the complexity of Sdc-1-dependent functions, which may affect cell adhesion, cell matrix-interactions, cytokine and chemokine activity, a modulation of proteolytic factors and expression changes in cell adhesion molecules (7, 9, 10). It is also conceivable that the murine forms of Sdc-1 may have influenced the functional status of endogenous human Sdc-1 in our assays. For example, in Sdc1-392 cells, nuclear localization of Sdc-1 appeared to be more prominent compared to the other forms (**Figure 2D**), which may have influenced E-cadherin expression and EMT (41). Also, the presence of uncleavable murine Sdc-1 could have resulted in increased compensatory shedding of endogenous Sdc-1, however, we could not find experimental evidence for this hypothesis (**Figure 2E**). Our data suggest that the migration phenotype may depend on cytoskeletal activity modulated by Sdc-1, whereas factors such as proteolysis and homotypic cell-cell adhesion may be of higher relevance for the invasion phenotype. Furthermore, in the matrigel invasion assay, Sdc-1-dependent interactions with this basement-membrane like extracellular matrix are of relevance, whereas this matrix was absent in the cell motility assay. It was demonstrated that, interacting with focal adhesions, Sdc-1 modulates regeneration of the tumor cell cytoskeleton in a rho-GTPase-dependent manner (42). Studies on MDA-MB-231 breast cancer cells found an association between Sdc-1 and Rho-GTPase in the regulation of cell motility, and in contrast to HeLa cells, MDA-MB-231 cell motility was increased *via* the Rho signaling pathway upon pathway upon Sdc-1 depletion (24). Moreover, data in an experimental model of lung injury has demonstrated that exosomes enriched in Sdc-1 ameliorate lung edema and inflammation *via* a mechanism that involves Rho-kinase signaling and cytoskeletal restructuring (43). In addition, calcification-independent vascular effects of osteoprotegerin have been ascribed to an activation of Sdc-1, and included osteoprotegerin-dependent activation of Rho kinase (44). Clinically, different functions of Sdc-1 between cervical and breast cancer have been observed. Whereas high Sdc-1 expression in mammary carcinoma is related to poor prognosis (32, 45), low Sdc-1 expression in cervical carcinoma is associated with poor differentiation and poor prognosis (6, 13). We showed that in HeLa cells, soluble Sdc-1 overexpression leads to changes in Rho B localization (**Figure 4C**). Since Sdc-1 and Rho-Kinases regulate cell motility, we suggest soluble Sdc-1 placing Rho B into a different activated status, affecting a change of localization to cell-cell borders. Different forms of Rho Kinases need to be relocated when they change from inactive cytoplasmatic form to activated plasma membrane form (42, 46). The involvement of Rho kinases in changes of cell-cell adhesion (42, 46) and the participation of Sdc-1 in the formation of focal adhesions is well known (9).

Some caveats are associated with the present study. Our study focused on clinicopathological data and a model cell line-based *in vitro* analysis. In the clinicopathological datasets, healthy tissue and metastatic samples were limited, requiring cautious interpretation of some of our results. Regarding the mechanistic data, further xenograft studies could help to corroborate our results in a setting that includes the tumor microenvironment, and could expand our study to find out, e.g., how Sdc-1 in its different forms affects angiogenesis, a mechanism which could promote metastases of the cervical cancer cells. We did not analyse the glycosylation status of our cells, which may have acted as a confounder. However, previous work in breast cancer cells applying the same methodological approach had not revealed major changes in heparan sulfate structure (10). Furthermore, future studies could address if Sdc-1 can function as a co-receptor for Human Papilloma Viruses as an important pathogenetic mechanism in cervical cancer (47). Finally, the role of Sdc-1 in the progression to a malignant phenotype apparently depends not only on its expression in the tumor cells, but also in the stroma, providing a rationale for studies in a co-culture setting.

In conclusion, In HeLa cervical cancer cells, membrane-bound and soluble forms of Sdc-1 modulate cell proliferation, apoptosis, motility, and invasiveness. These observations suggest an important role for Sdc-1 in the progression of cervical cancer. The observation of decreased cell motility in Sdc-1 overexpressing HeLa cells (**Figure 4**) is consistent with clinical data on Sdc-1-dependent lymph node metastasis, as a previous study on 106 tissue specimens showed an inverse correlation between Sdc-1 expression in the primary site of cervical carcinomas and lymph node metastases (15). Therefore, reduced cell motility in Sdc-1 expressing cervical carcinoma cells may contribute to a reduction in metastatic behaviour. However, the function of Sdc-1 appears to be context-dependent, as we observed increased invasiveness of Sdc-1 WT and Sdc1-392 HeLa cells in the Matrigel invasion chamber assay (**Figure 3E**, see discussion above). Sdc-1 modulates cell motility in a Rho-GTPase-dependent manner. The membrane-bound and the soluble Sdc-1 can be assigned different functions, the detailed analysis of which appears worthwhile in future studies. Understanding the mechanisms by which Sdc-1 promotes these processes could help to better understand the behavior of cervical cancer and find specific therapeutic targets.

## Data Availability Statement

The original contributions presented in the study are included in the article/supplementary material. Further inquiries can be directed to the corresponding author.

## Author Contributions

KH performed the *in vitro* experiments and analyzed the data. NE-S performed flow cytometry. SI and BG supervised the data and provided the expertise on cell proliferation, apoptosis, invasion and migration assays analysis. LK provided the general support, co-supervised KH, and was involved in the data interpretation. MG conceived and coordinated the study. NE-S and MG performed database analysis, generated the figures and wrote the manuscript. All authors contributed to the article and approved the submitted version.

## Funding

This work was supported by EU H2020 MSCA-RISE project no. 645756 GLYCANC (to MG, SI) and the Deutsche Forschungsgemeinschaft DFG GO 1392/8-1 (to MG) and GR4743/5-1 (to BG).

## Conflict of Interest

The authors declare that the research was conducted in the absence of any commercial or financial relationships that could be construed as a potential conflict of interest.

## Publisher’s Note

All claims expressed in this article are solely those of the authors and do not necessarily represent those of their affiliated organizations, or those of the publisher, the editors and the reviewers. Any product that may be evaluated in this article, or claim that may be made by its manufacturer, is not guaranteed or endorsed by the publisher.
